# Supplementary material for: BAFopathies’ DNA methylation epi-signatures demonstrate diagnostic utility and functional continuum of Coffin–Siris and Nicolaides–Baraitser syndromes
Source: Nat Commun. 2018 Nov 20;9:4885. doi: 10.1038/s41467-018-07193-y (PMC6244416; doi:10.1038/s41467-018-07193-y)
Supplement: Supplementary file 1 — Supplementary Information [file 41467_2018_7193_MOESM1_ESM.pdf]

Supplementary Information

BAFopathies' DNA methylation epi-signatures demonstrate diagnostic utility and functional continuum of Coffin-Siris and Nicolaides-Baraitser syndromes

Erfan Aref-Eshghi[1,2], Eric G. Bend[3], Rebecca L. Hood[4], Laila C. Schenkel[1,2], Deanna Alexis Carere[2], Rana Chakrabarti[5], Sandesh C.S. Nagamani[6], Sau Wai Cheung[6], Philippe M. Campeau[7], Chitra Prasad[5], Victoria Mok Siu[5], Lauren Brady[8], Mark A. Tarnopolsky[8], David J. Callen[8], A. Micheil Innes[9], Susan M. White[10], Wendy S. Meschino[11], Andrew Y. Shuen[5], Guillaume Paré[12], Dennis E. Bulman[4], Peter J. Ainsworth[1,2], Hanxin Lin[1,2], David I. Rodenhiser[5,13], Raoul C. Hennekam[14], Kym M. Boycott[4], Charles E. Schwartz[15], Bekim Sadikovic[1,2*]

[1] Department of Pathology and Laboratory Medicine, Western University, London, ON, Canada

[2] Molecular Genetics Laboratory, Molecular Diagnostics Division, London Health Sciences Centre, London, ON, Canada

[3] Prevention Genetics, Marshfield, WI, USA

[4] Children's Hospital of Eastern Ontario Research Institute, University of Ottawa, Ottawa, ON, Canada

[5] Children's Health Research Institute, London, ON, Canada

[6] Department of Molecular and Human Genetics, Baylor College of Medicine, Houston, TX, USA

[7] Department of Pediatrics, University of Montreal, Montreal, QC, Canada

[8] Department of Pediatrics, McMaster University, Hamilton, ON, Canada

[9] Department of Medical Genetics, Alberta Children's Hospital Research Institute for Child and Maternal Health, University of Calgary, Calgary, AB, Canada

[10] Department of Paediatrics, University of Melbourne, Melbourne, Australia

[11] Genetics Program, North York General Hospital, Toronto, ON, Canada

[12] Department of Pathology and Molecular Medicine, McMaster University, Hamilton, ON, Canada

[13] Department of Pediatrics, Biochemistry and Oncology, Western University, London, ON, Canada

[14] Department of Pediatrics, Academic Medical Center, University of Amsterdam, Amsterdam, Netherlands

[15] Greenwood Genetic Center, Greenwood, SC, USA

*Corresponding author: Bekim Sadikovic, Ph.D., DABMGG, FACMG; Department of Pathology and Laboratory Medicine, Victoria Hospital, London Health Sciences Centre; 800 Commissioner's Road E, B10-104, London, ON, Canada, N6A 5W9; Phone: (519) 665-8500 Ext. 53074; E-mail: Bekim.Sadikovic@lhsc.on.ca

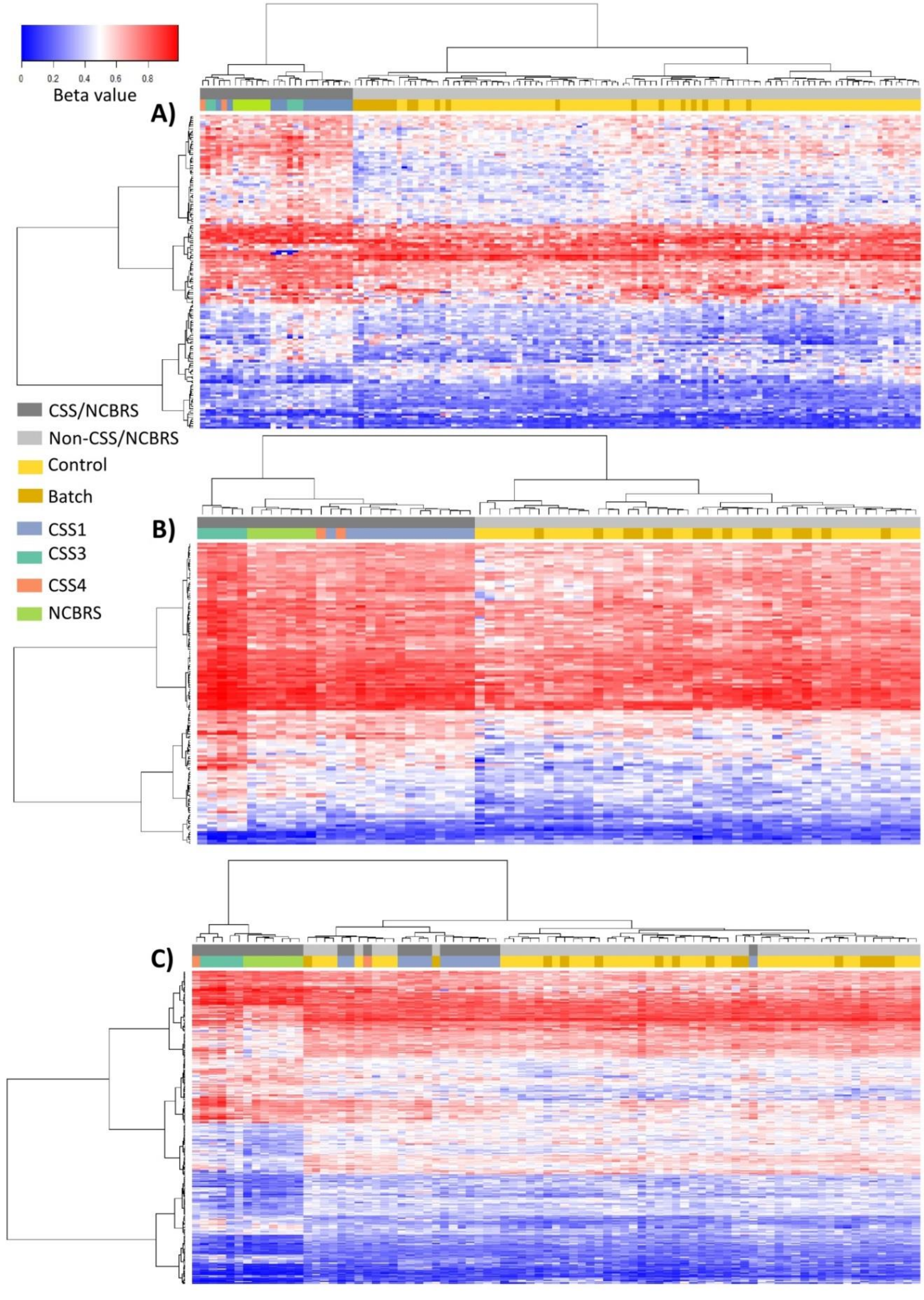

Supplementary Figure 1- Clustering analysis using three probe-sets identified for CSS1, CSS3 and NCBRS

This figure illustrates the same clustering analysis performed in figures 1 and 2 in the main text, after inclusion of a random set of other non-CSS/NCBRS DD/ID patients from the same batch as CSS/NCBRS. These new samples are shown using the brown pane and as seen, none are clustered with the CSS/NCBRS groups. The objective here was to show that any observed pattern in these plots is not due to batch effect or a profile existing in all DD/ID cases.

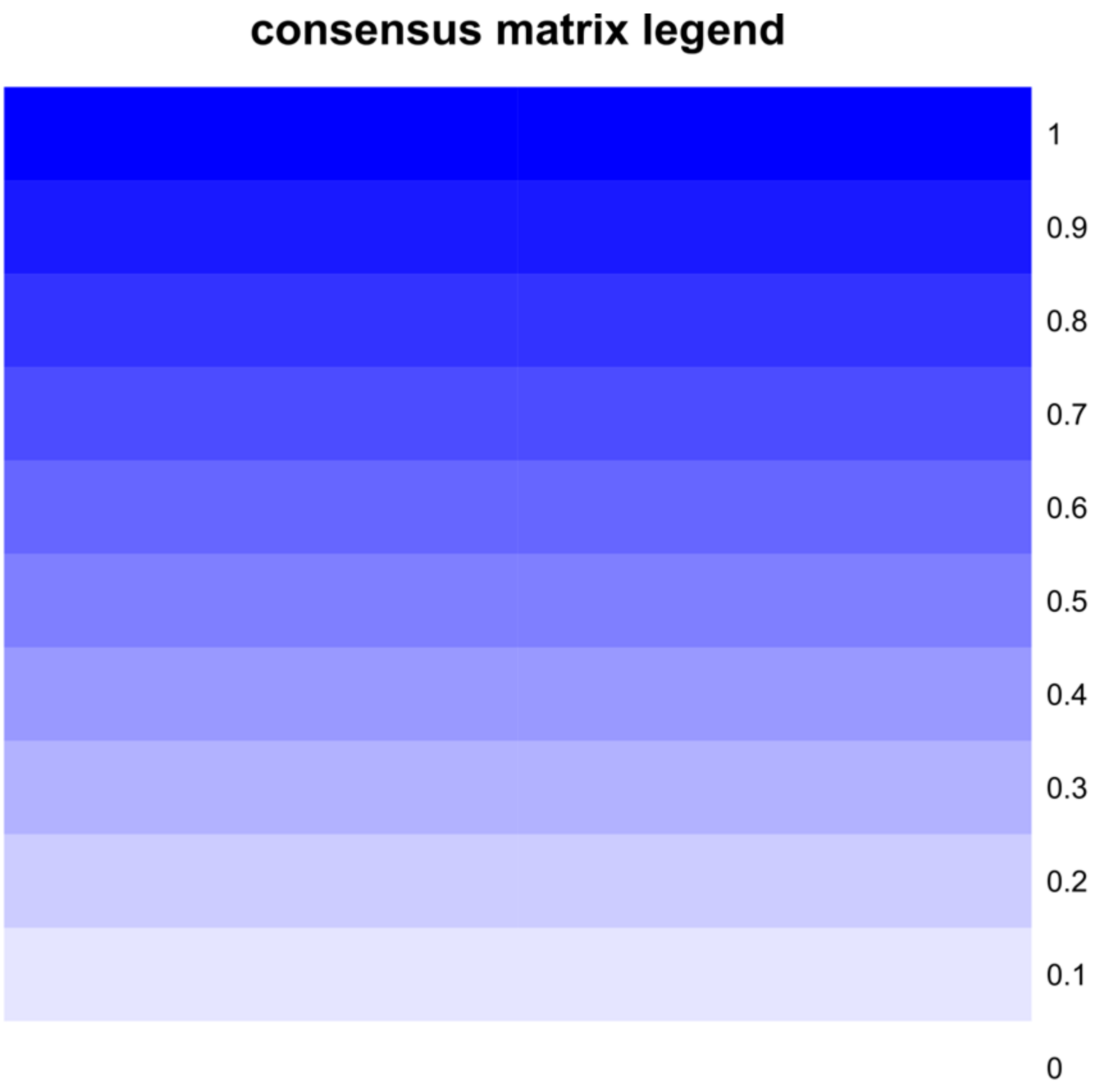

Supplementary Figure 2- The color scale of the consensus matrix

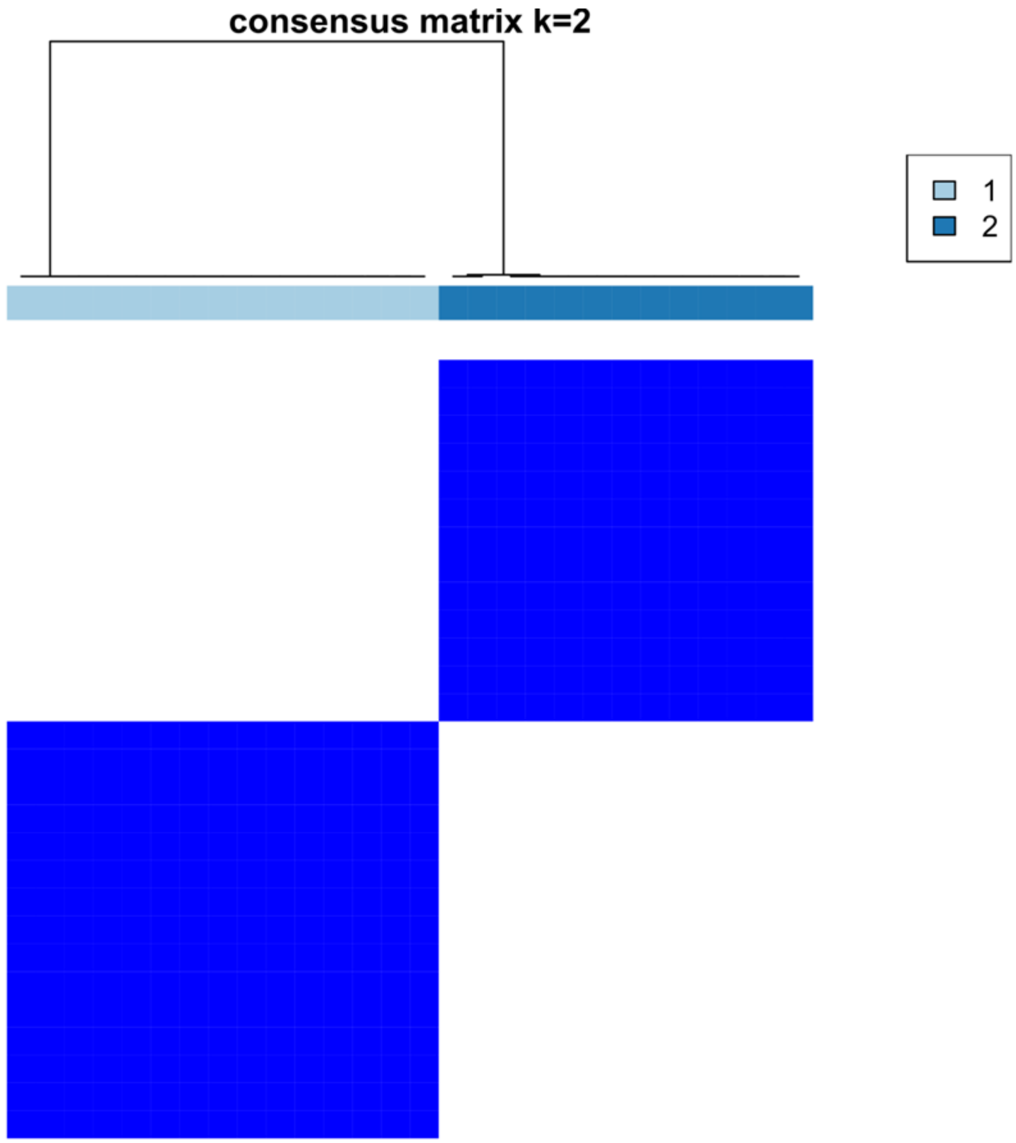

Supplementary Figure 3- Consensus clustering at k=2

The order of the samples in this plot and the following clustering plots (Figures S3-11) remains constant as shown in Supplementary Figure 14. For interpretations of Figures S3-11 see Figure 3 in the manuscript.

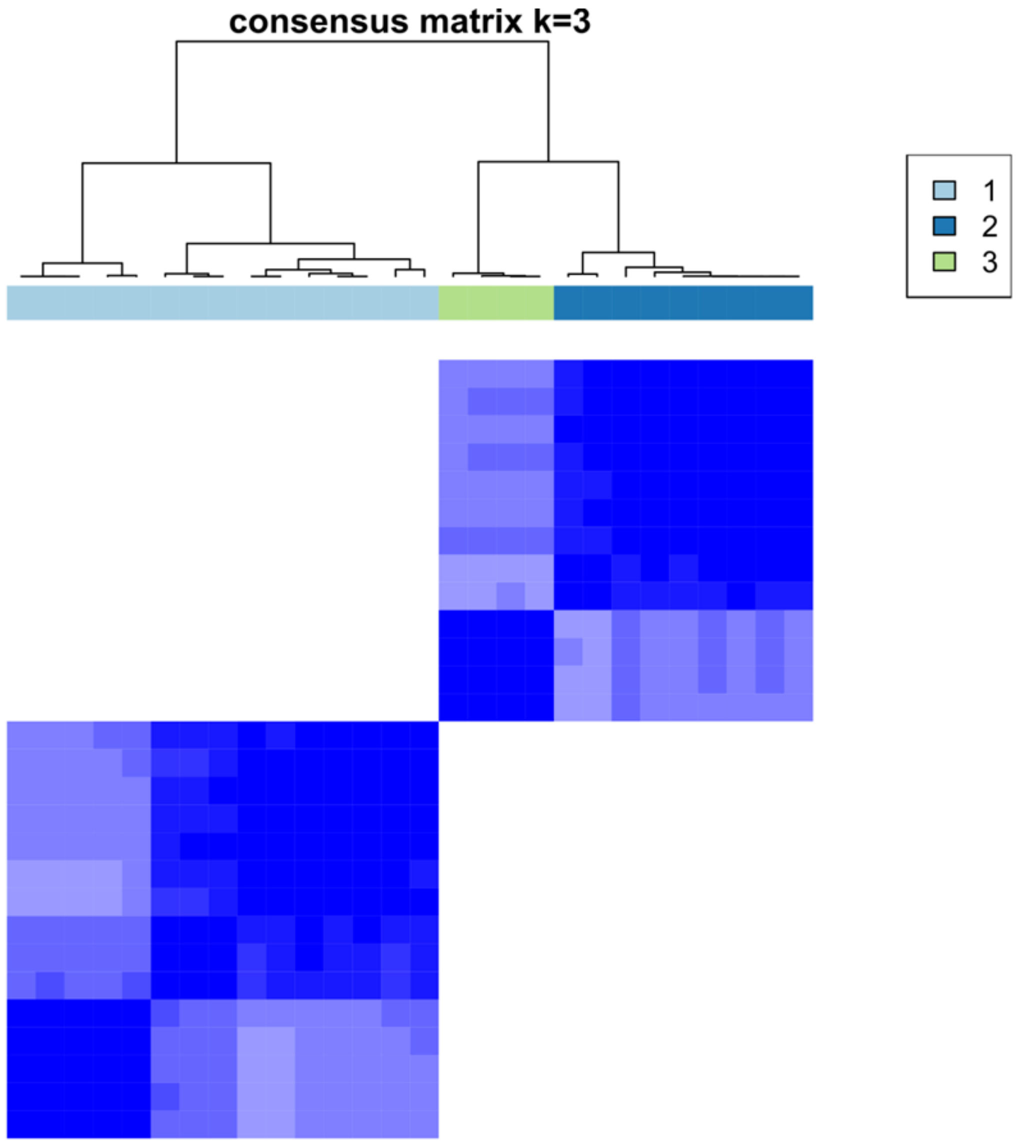

Supplementary Figure 4- Consensus clustering at k=3

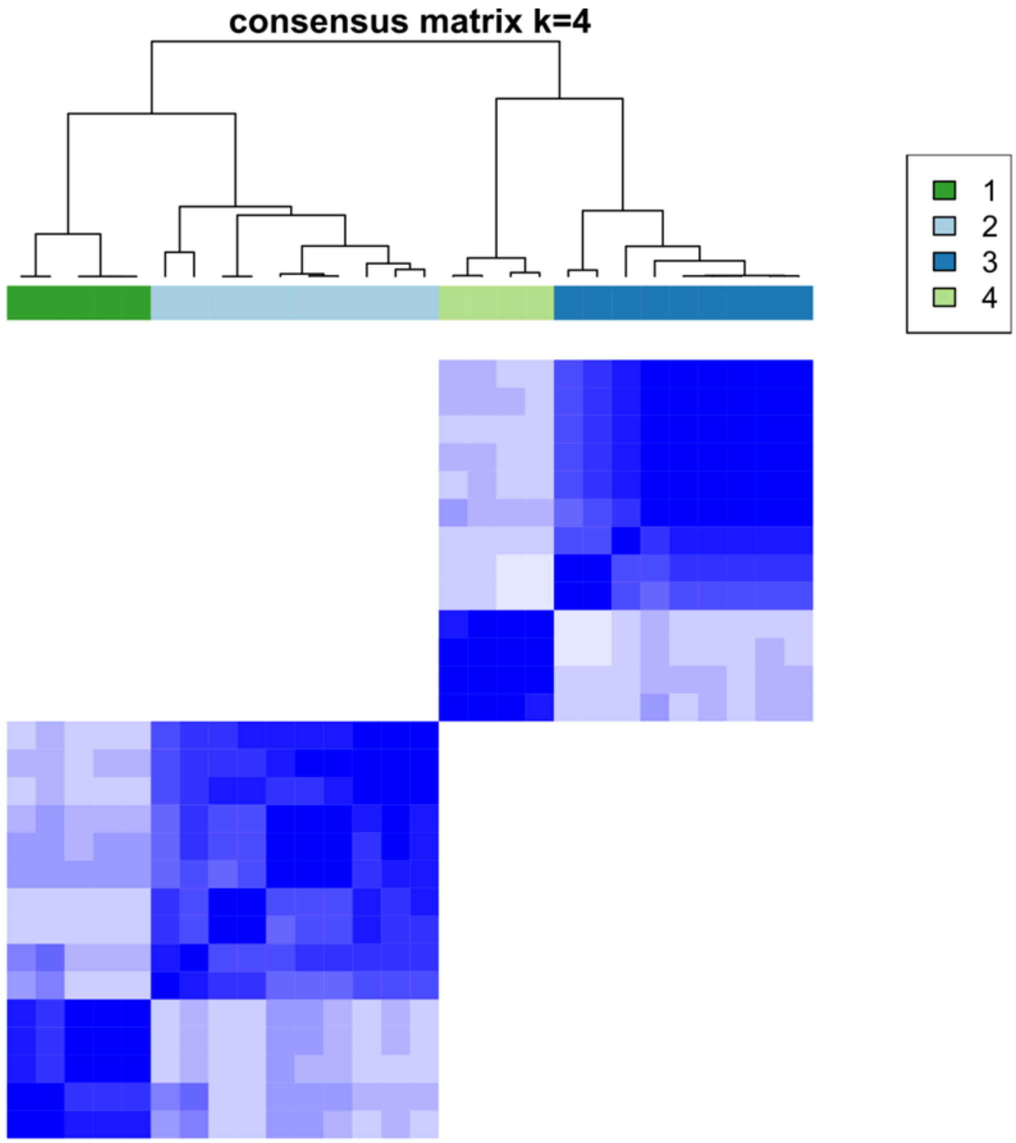

Supplementary Figure 5- Consensus clustering at k=4

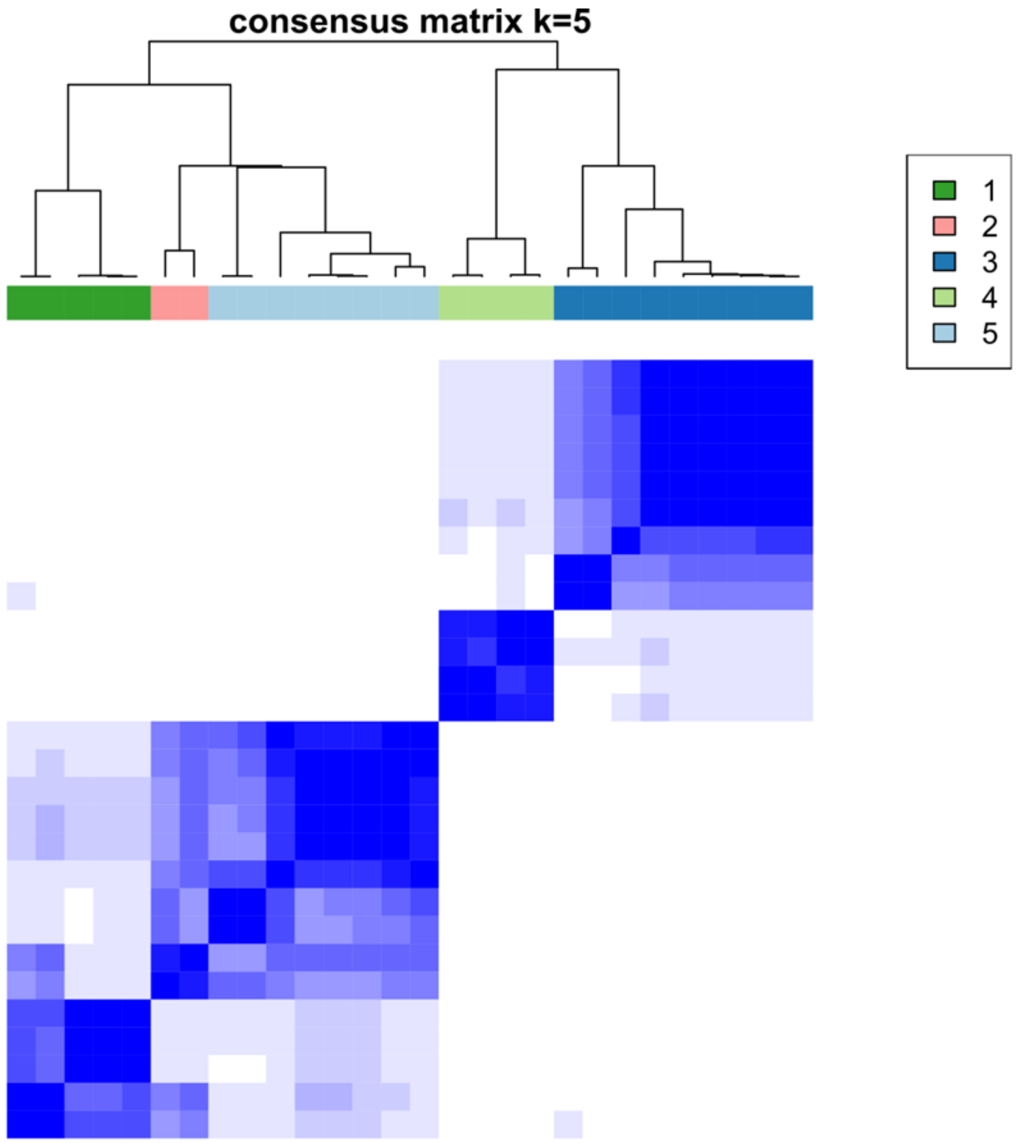

Supplementary Figure 6- Consensus clustering at k=5

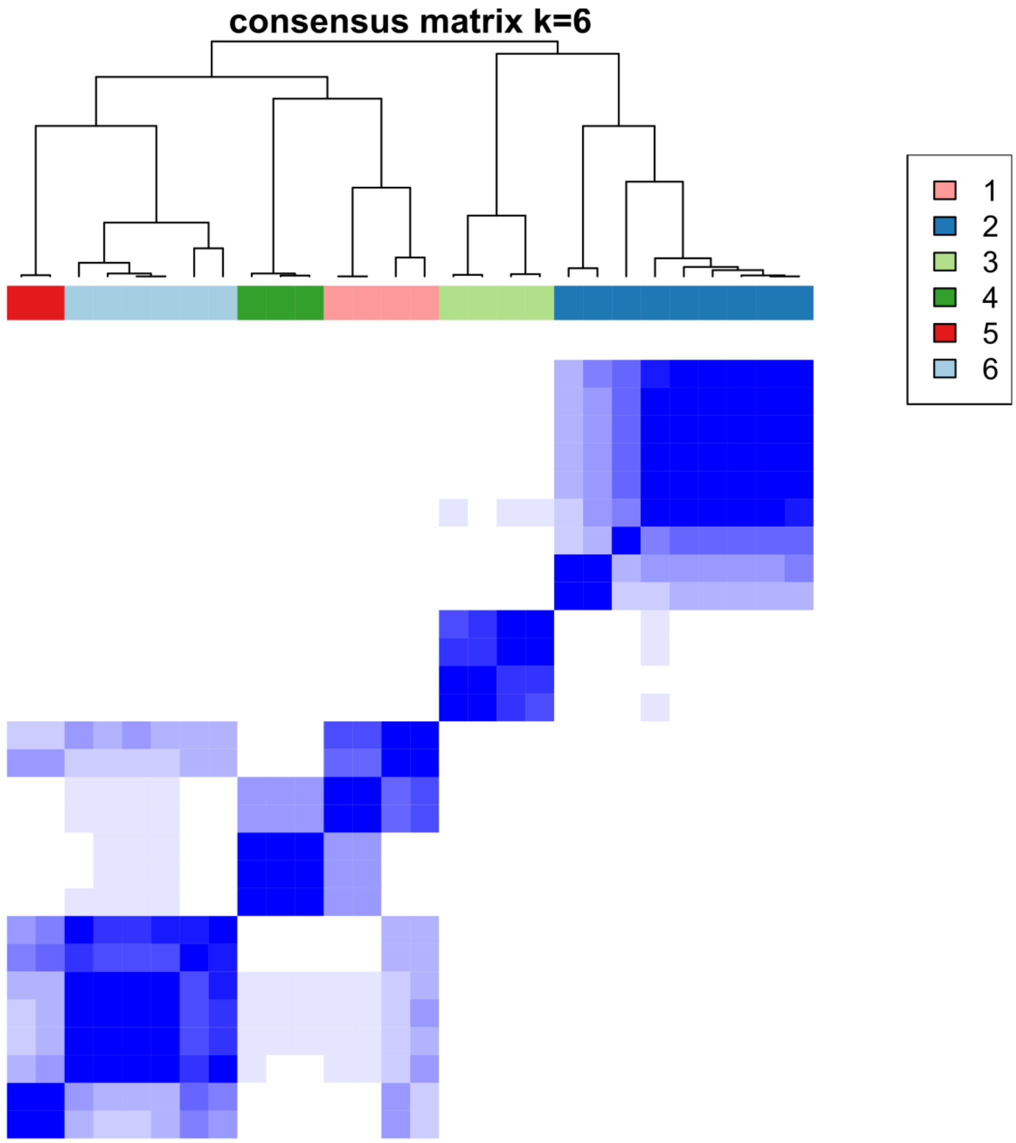

Supplementary Figure 7- Consensus clustering at k=6

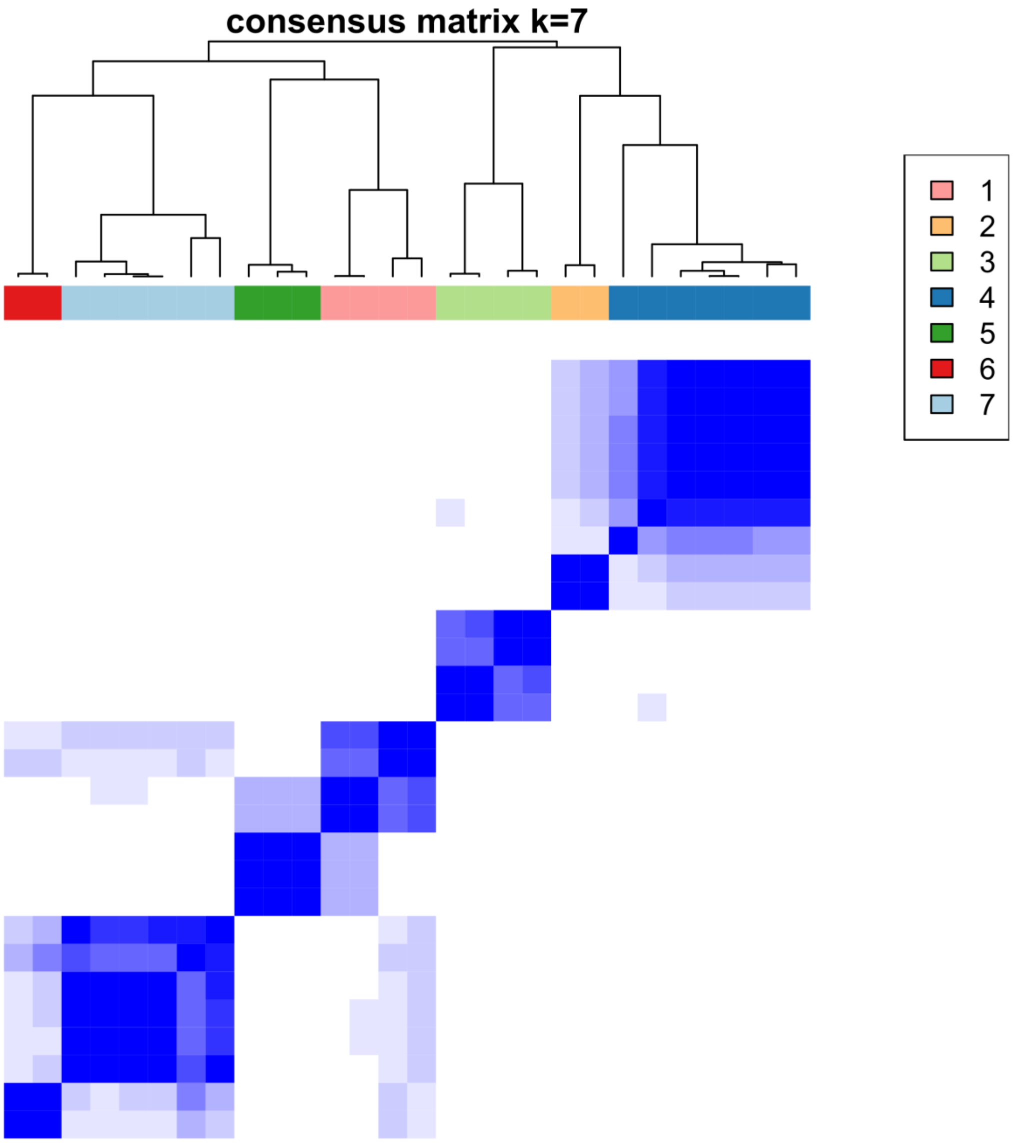

Supplementary Figure 8- Consensus clustering at k=7

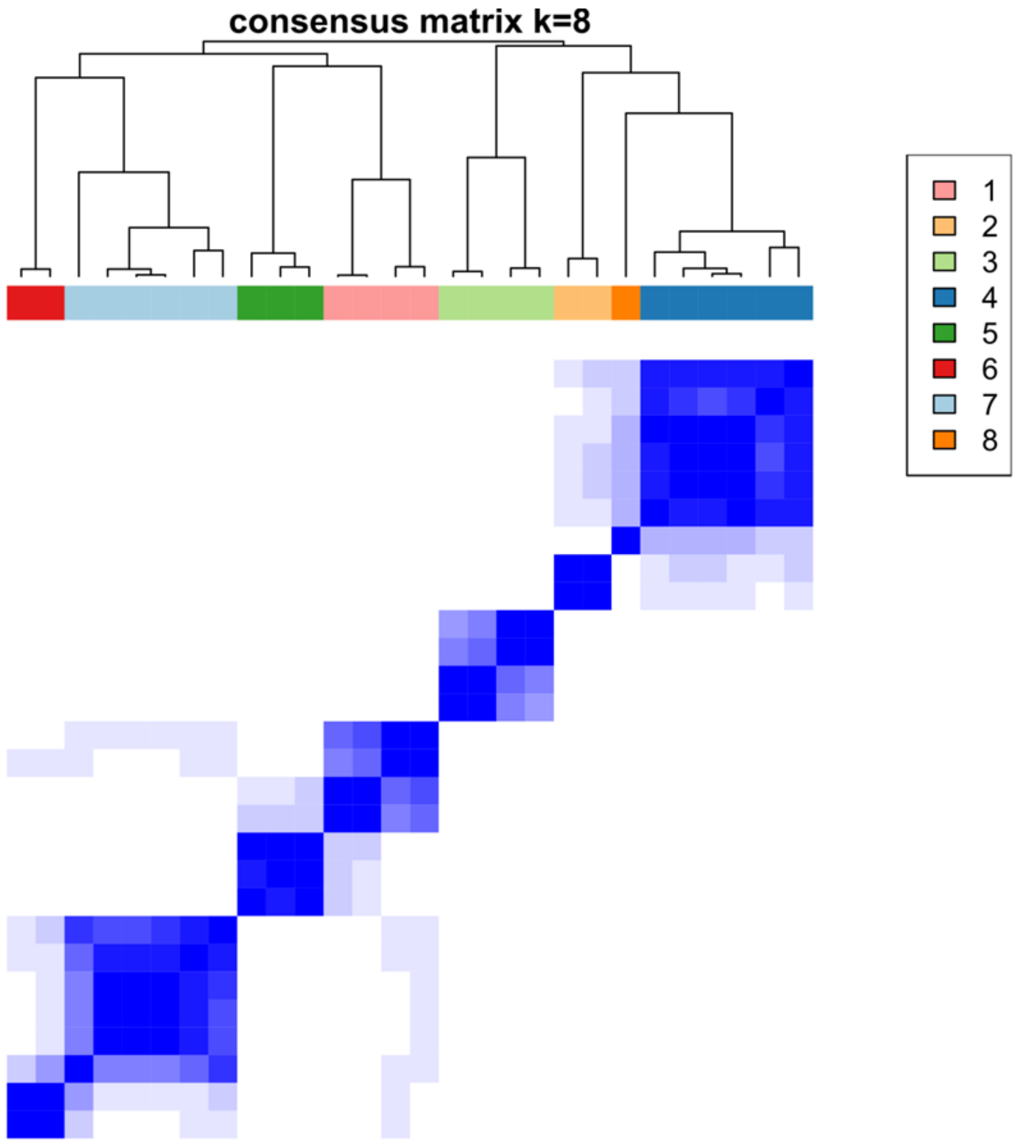

Supplementary Figure 9- Consensus clustering at k=8

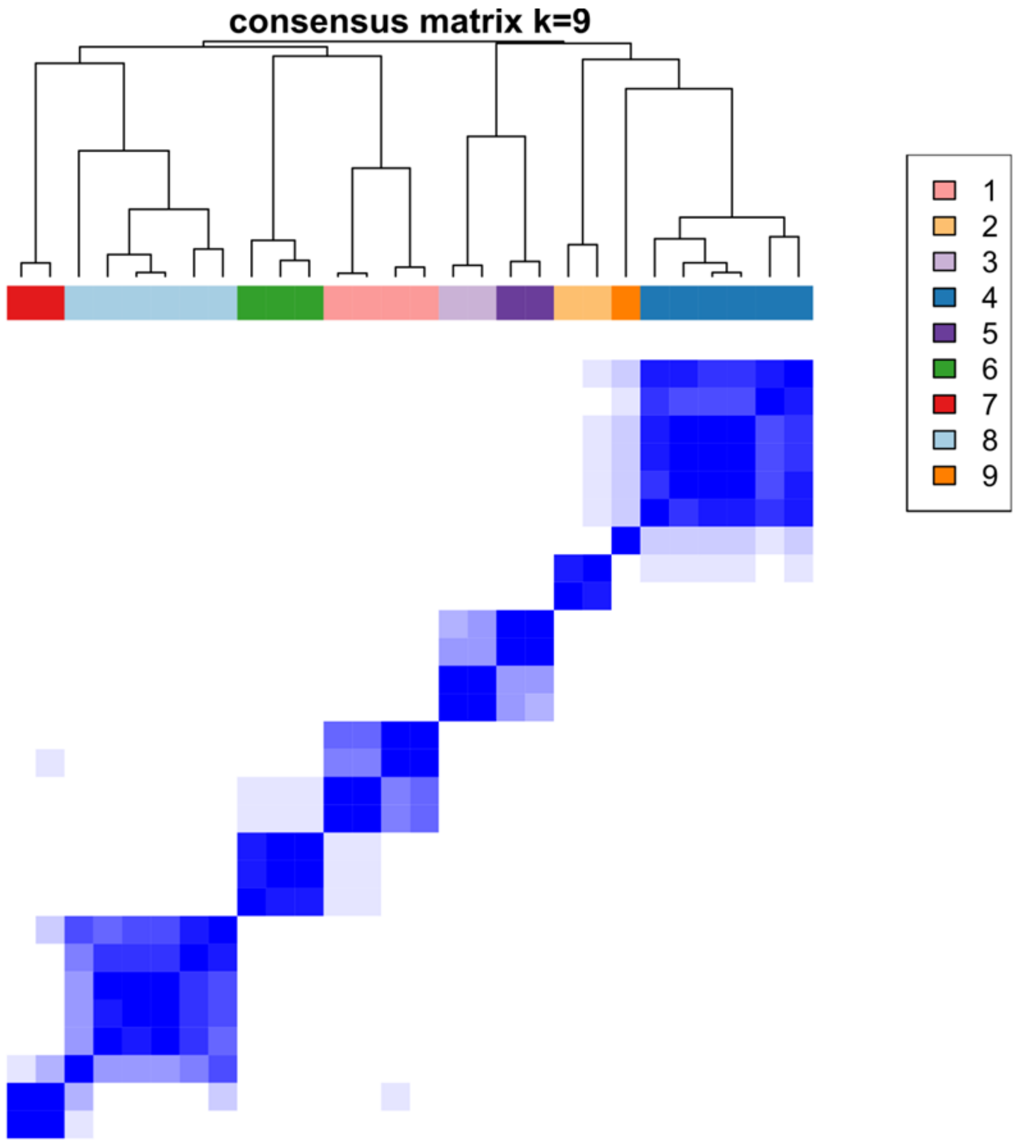

Supplementary Figure 10- Consensus clustering at k=9

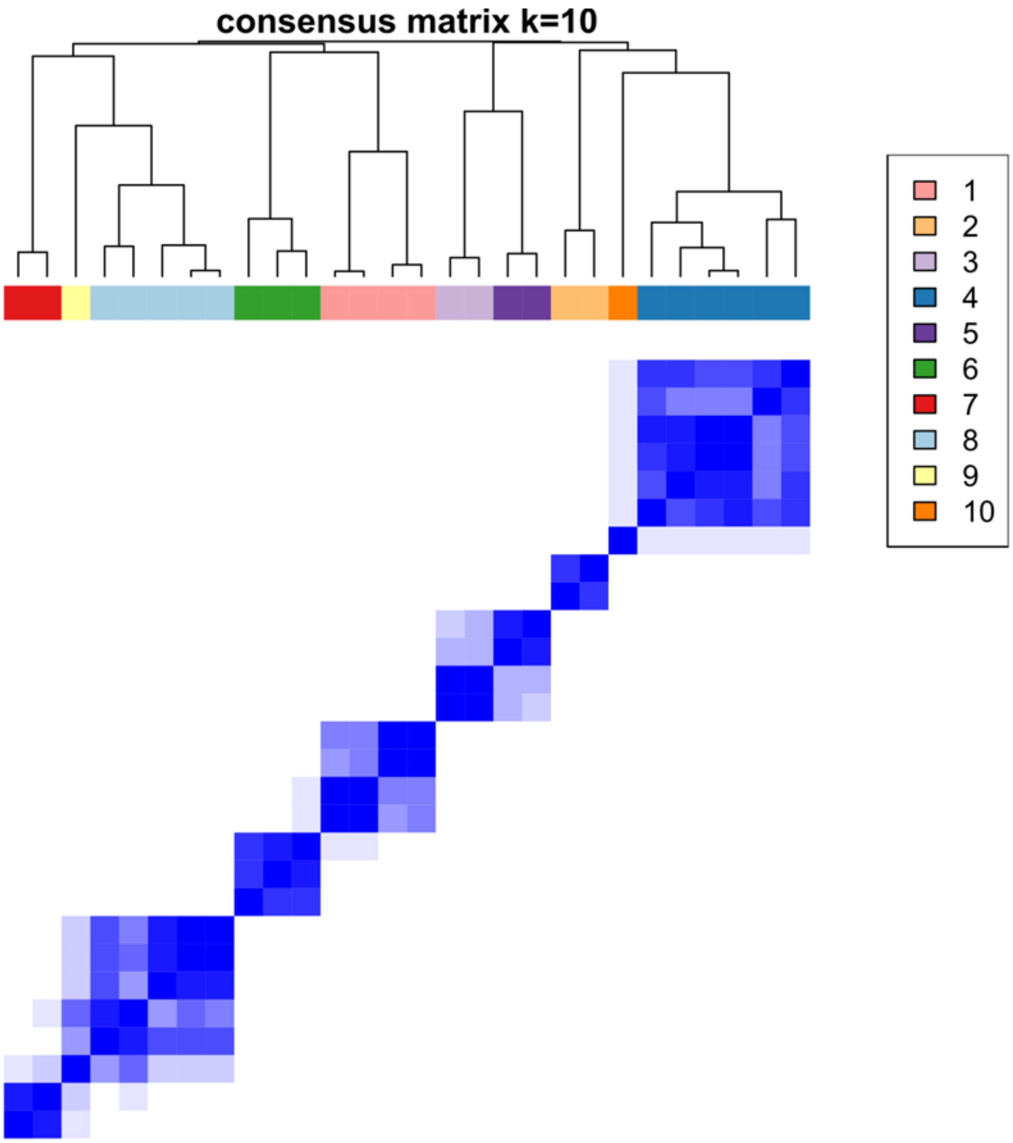

Supplementary Figure 11- Consensus clustering at k=10

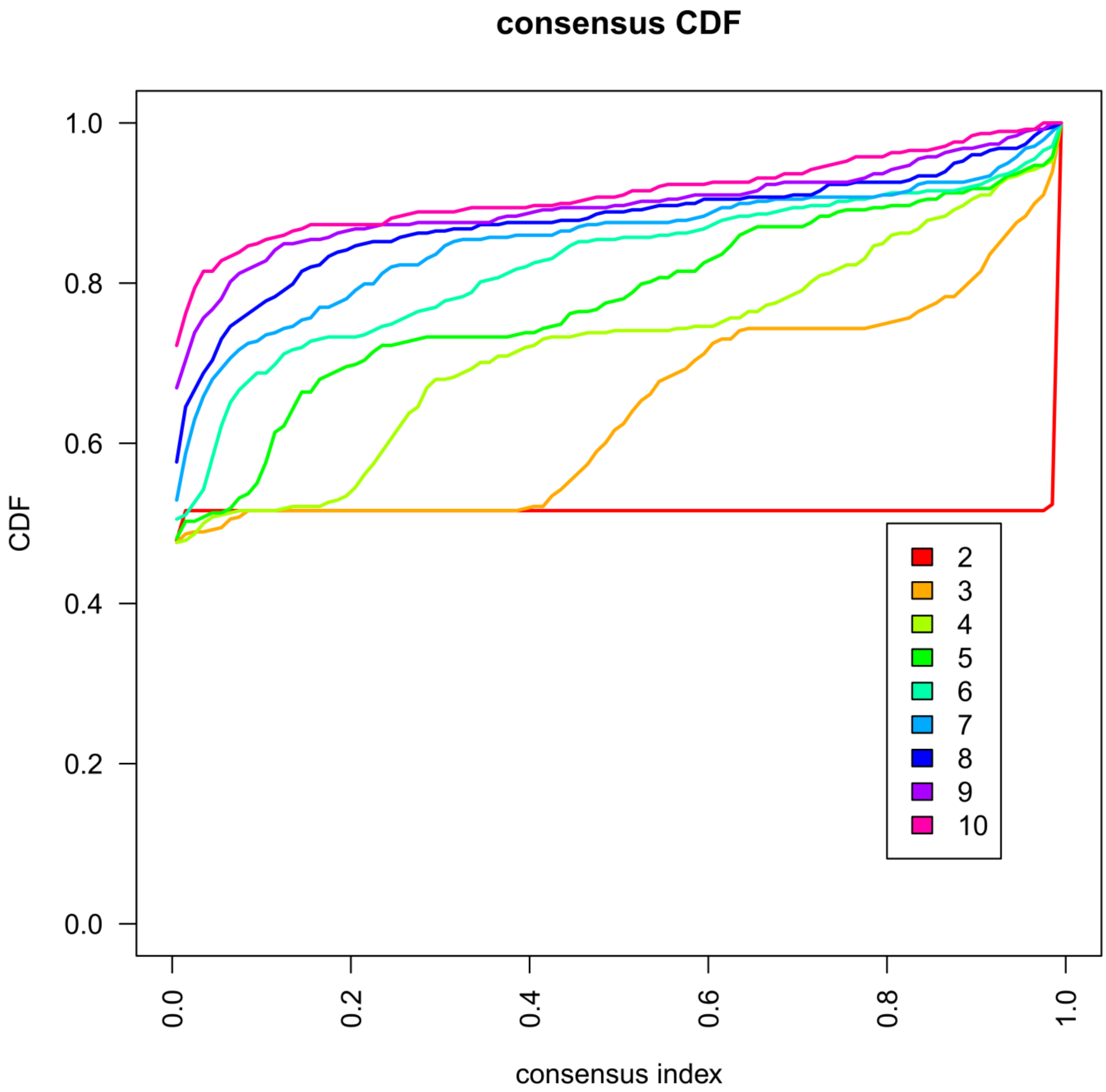

Supplementary Figure 12- Consensus cumulative distribution function (CDF) plot

This figure illustrates the CDF plot of the consensus matrix for each k (stratified by colors). In any curve of a consensus matrix, the lower left portion represents samples that rarely clustered together, the upper right portion represents those always clustered together, and the middle segment represent those with ambiguous assignments in different iterations. The proportion of ambiguous clustering (PAC) measure is defined as the CDF value of the sample pairs with consensus index=1 minus CDF value for those close to 0 (top right vs. lower left, e.g. 0.1 and 0.9). A low PAC value indicates a flat middle segment and a low rate of discordance across permuted clustering runs. In the figure above, the PAC measure for clustering at k=2 is the smallest (almost zero), suggesting that k=2 is the most optimal cluster count in this analysis.

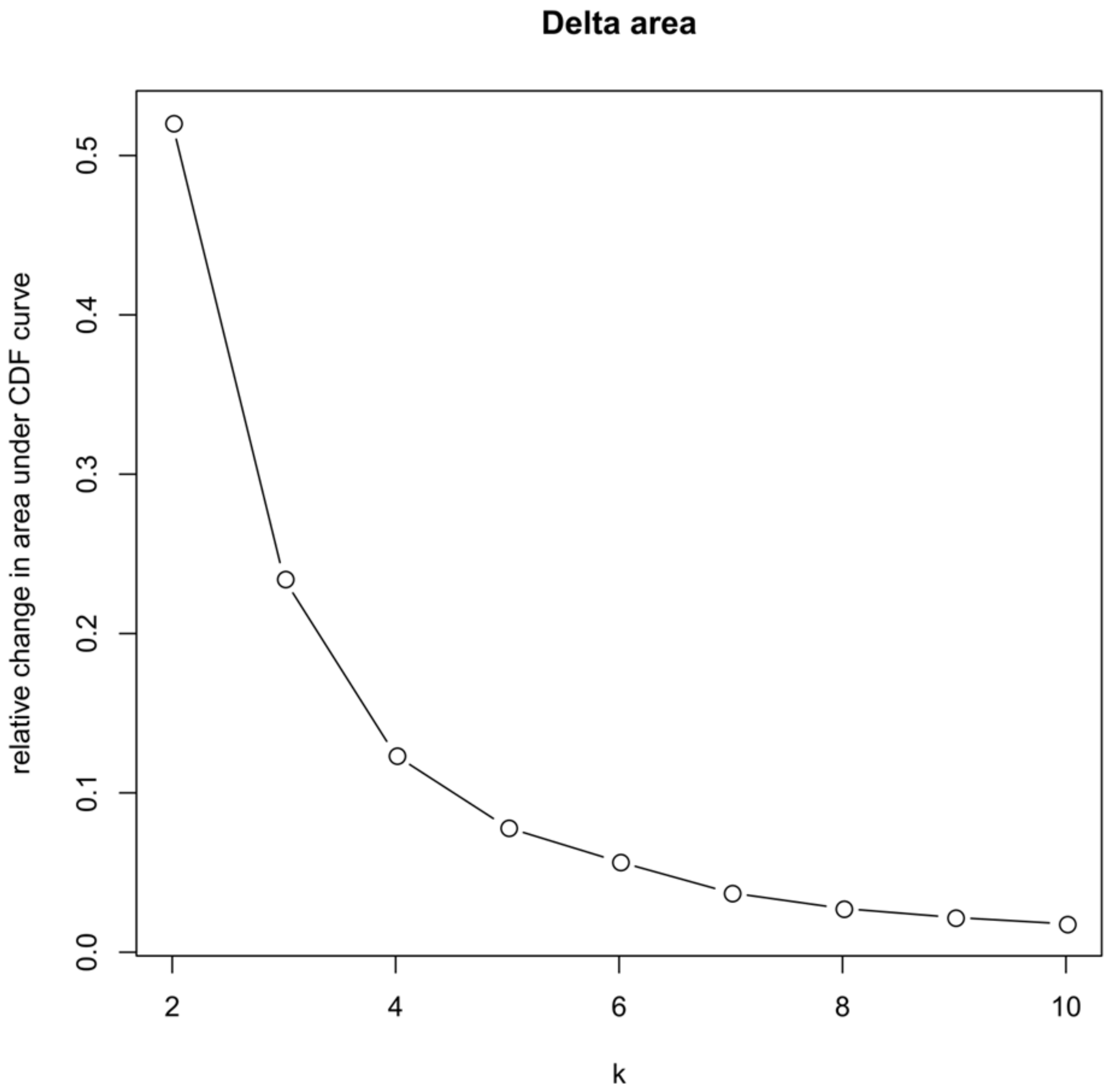

Supplementary Figure 13- Delta area plot

This figure shows the relative increase in the clustering consensus following a change from k-1 to k. The k providing the biggest change in the consensus represents the most optimum cluster count. In this plot, after k=2, none of the other k's result in a considerable increase in the consensus.

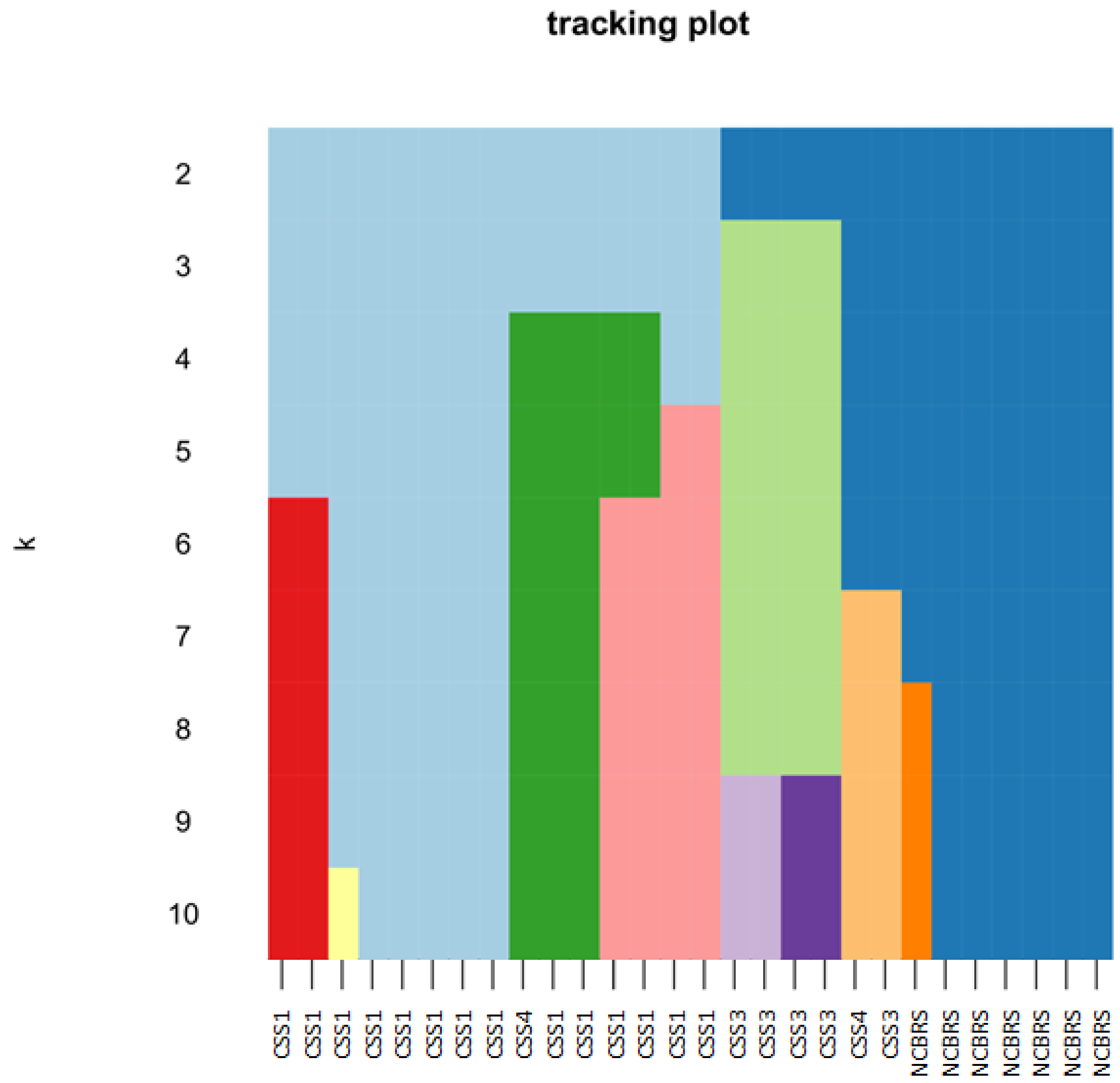

Supplementary Figure 14- Tracking plot

This figure shows the change in cluster assignments from k=2 to k=10 for every sample. Colors correspond to the colors of the clusters shown in the original clustering plots (Figures S3-11).

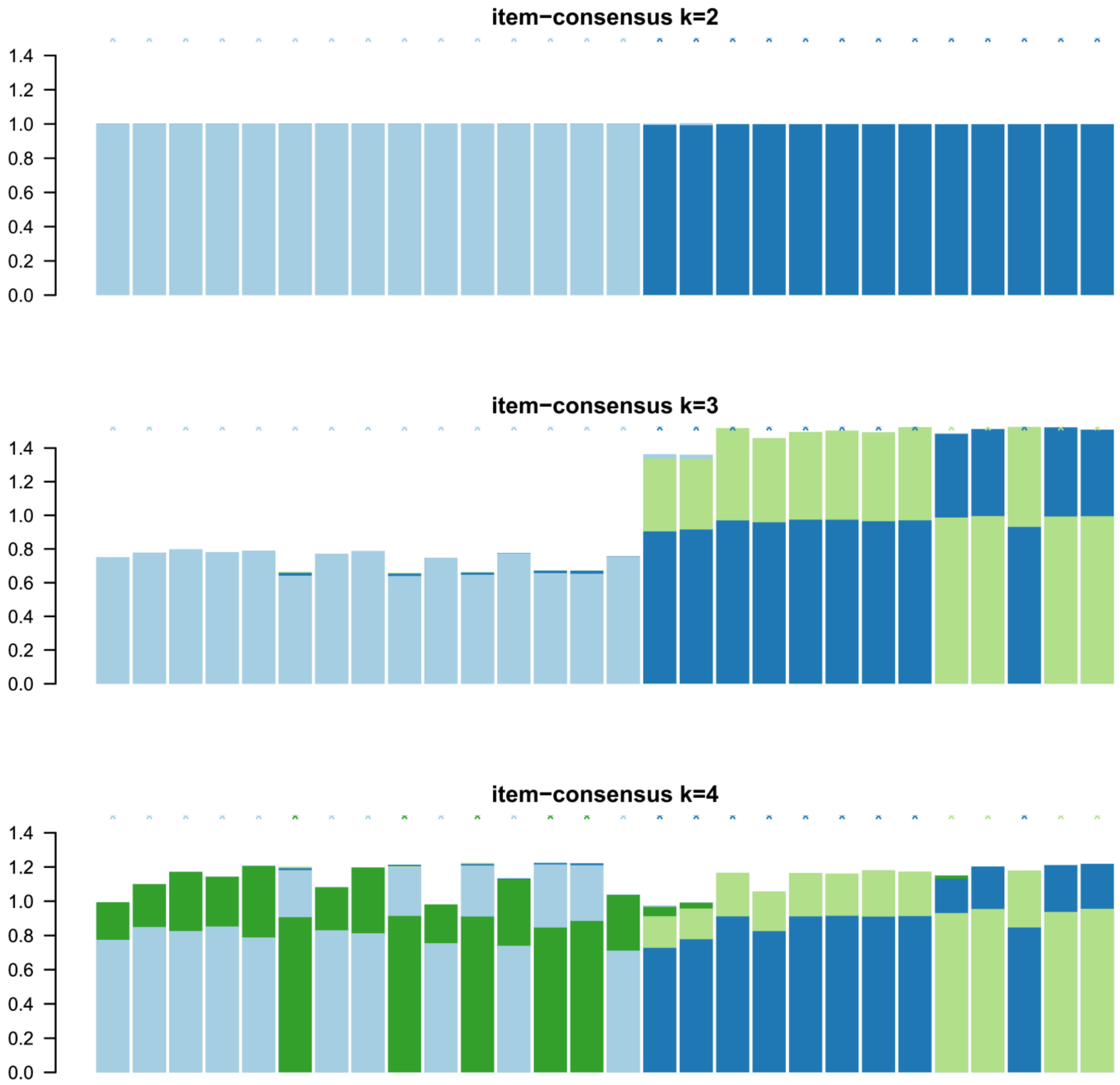

Supplementary Figure 15- Item consensus plots from k=2 to k=4

For interpretation see Figure 3 in the manuscript.

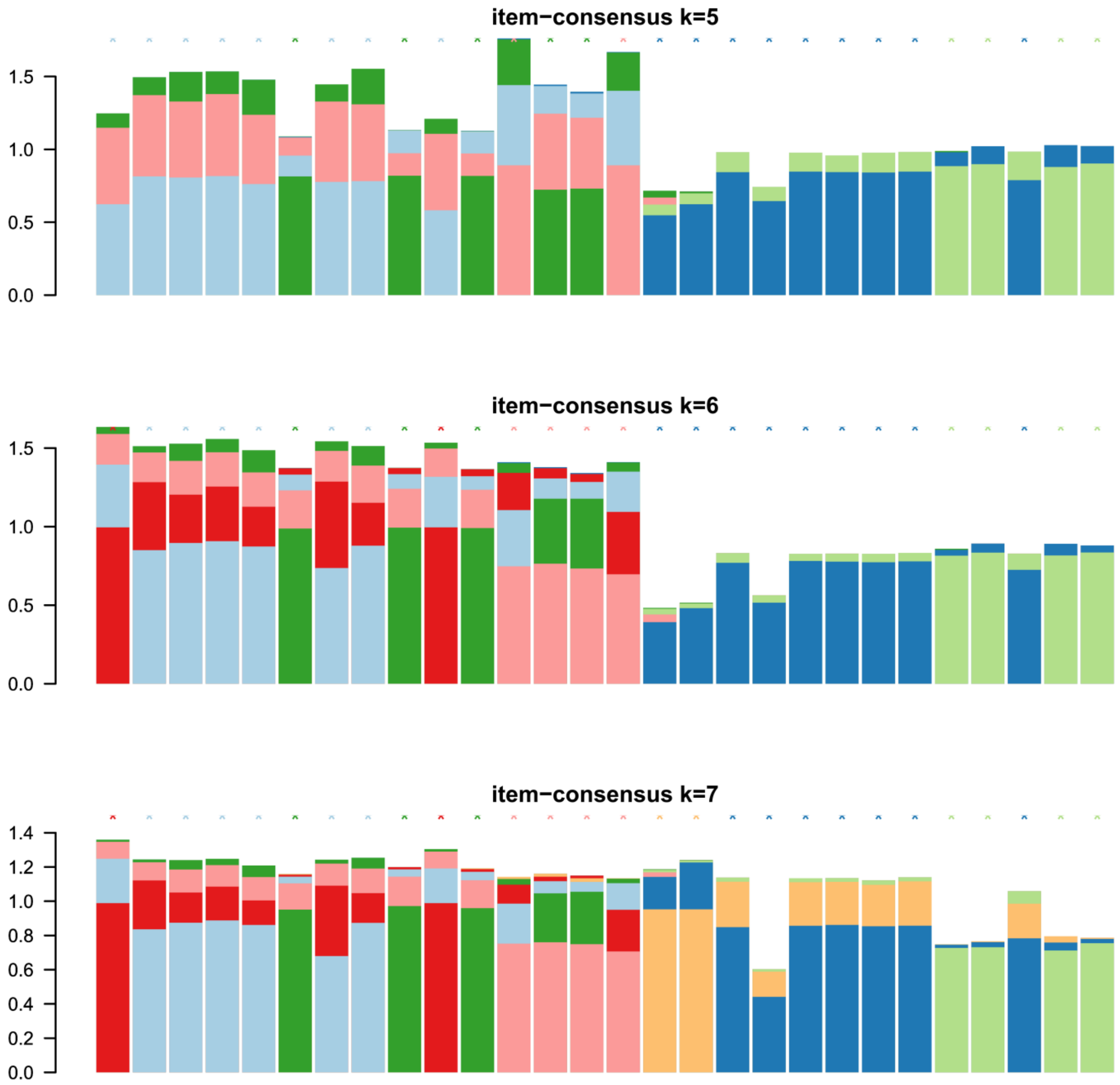

Supplementary Figure 16- Item consensus plots from k=5 to k=7

For interpretation see Figure 3 in the manuscript.

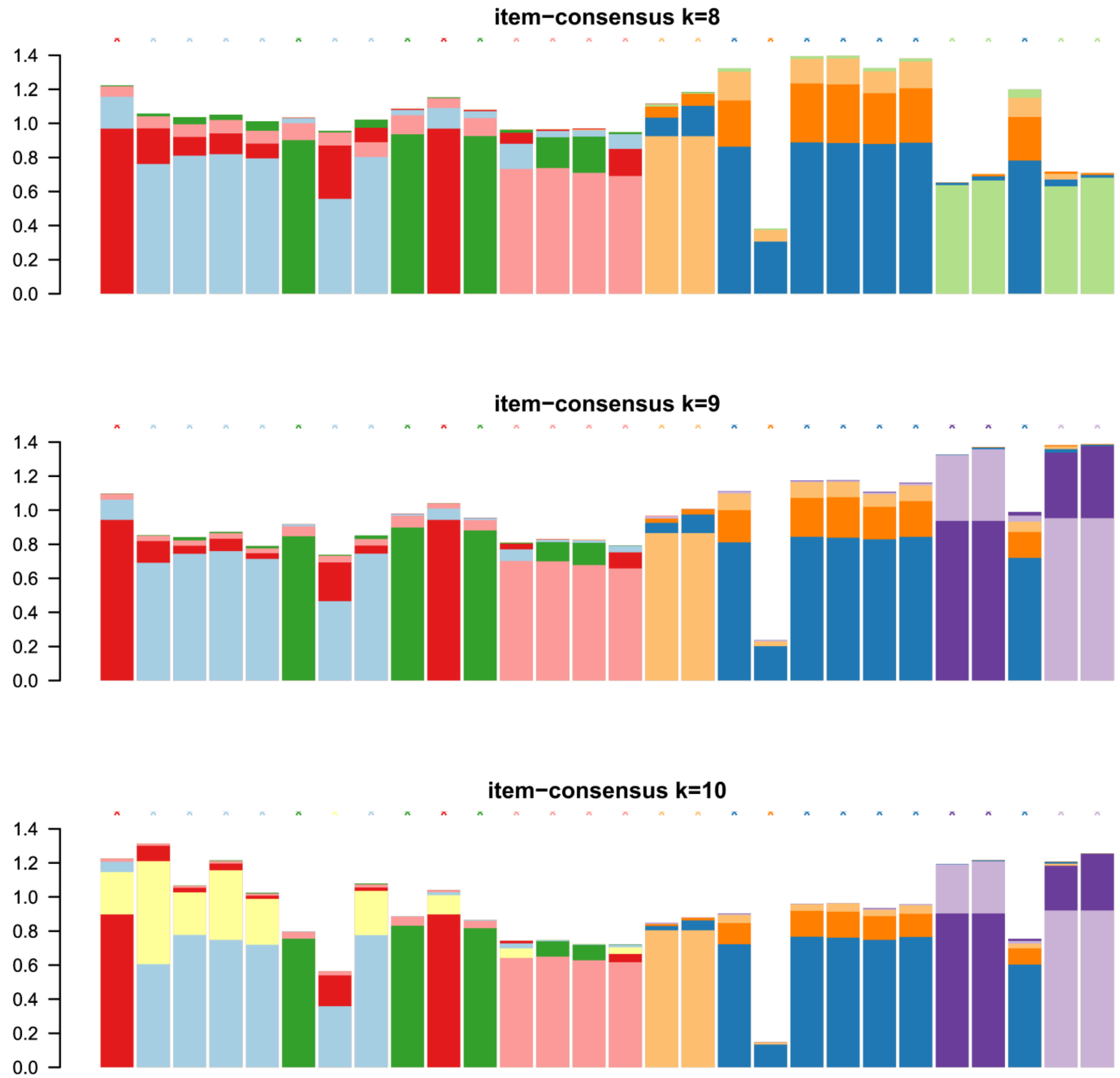

Supplementary Figure 17- Item consensus plots from k=8 to k=10

For interpretation see Figure 3 in the manuscript.

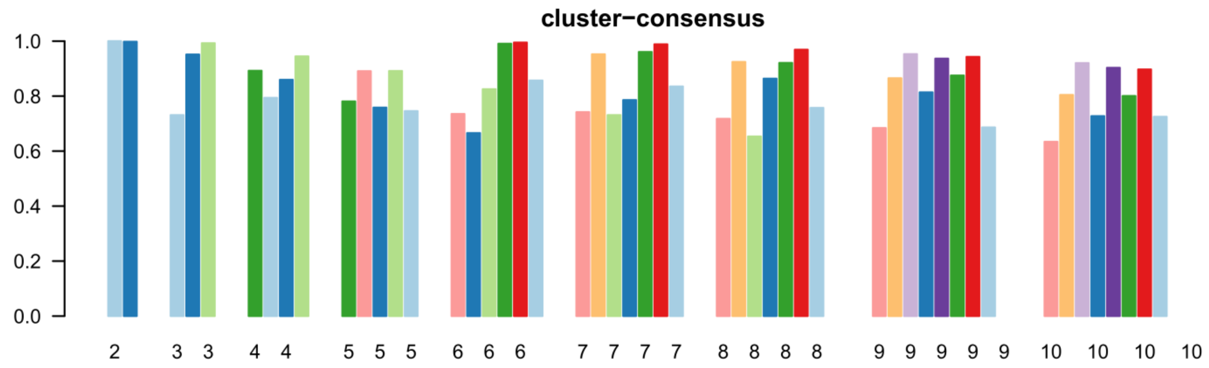

Supplementary Figure 18- Cluster consensus plot

This image shows the cluster-consensus value (0-1) for different clusters generated at each k, representing the proportion of times every sample in a cluster was grouped with members of its own cluster in a total of 1,000 clustering iterations. The bar colors correspond to the original cluster colors in Figures S3-11. High consensus values for each cluster represent higher stability. As seen, only k=2 generates clusters with all consensus values >0.99. The consensus is reduced significantly at k>2.

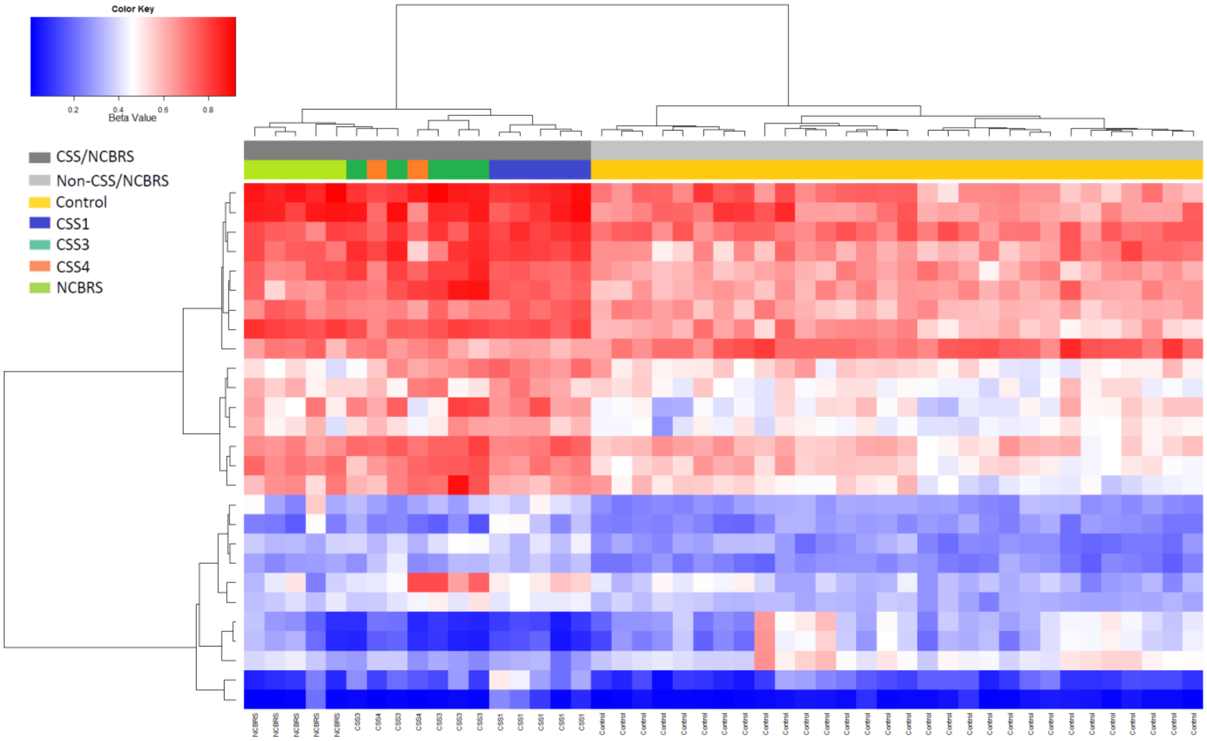

Supplementary Figure 19- Re-analysis using five randomly selected CSS1 subjects

To address the question of whether the relatively large CSS1 sample size has resulted in the generation of a more reliable probe list, and thus, lack of distinction across different CSS/NCBRS subtypes. We randomly selected five CSS1 cases and compared them with 30 matched controls, exactly as conducted in the manuscript. The sample size of five was chosen to make the sample count equal to that in CSS3 (five cases and 30 controls). This analysis identified 27 probes which perform equally well as the initial 146 CSS1 probes in placing all BAFopathies into one cluster, indicating that our initial observation of CSS1 having a profile that is shared across all BAFopathies is not influenced by the relatively larger sample size of CSS1.

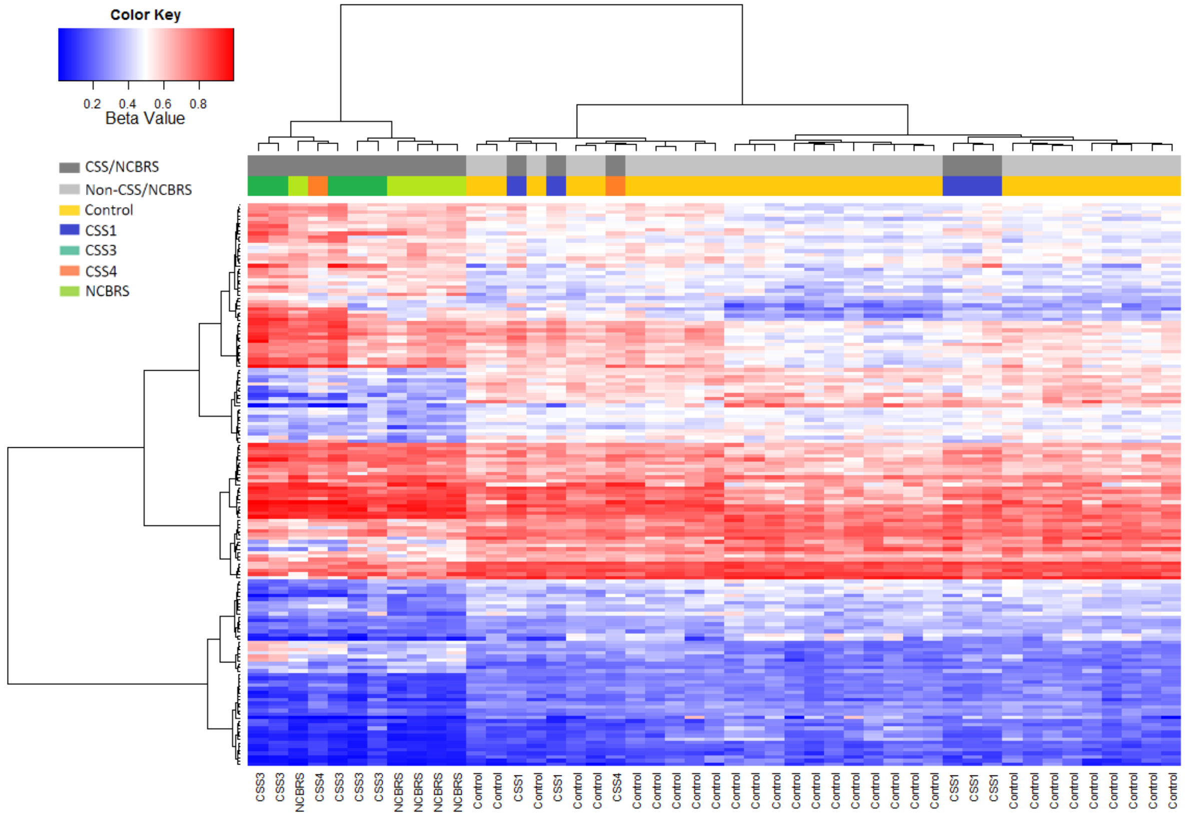

Supplementary Figure 20- Re-analysis using five randomly selected NCBRS subjects

A similar analysis to that in Supplementary Figure 19 was performed for NCBRS cases. As expected, the identified probe count was reduced to 157 from the initial 365. However, this did not change their performance in the clustering analysis, and similar to the initially identified probes, this experiment clustered controls and CSS1 into one cluster, CSS3 and NCBRS into the other, and split the two CSS4 subjects between the two.

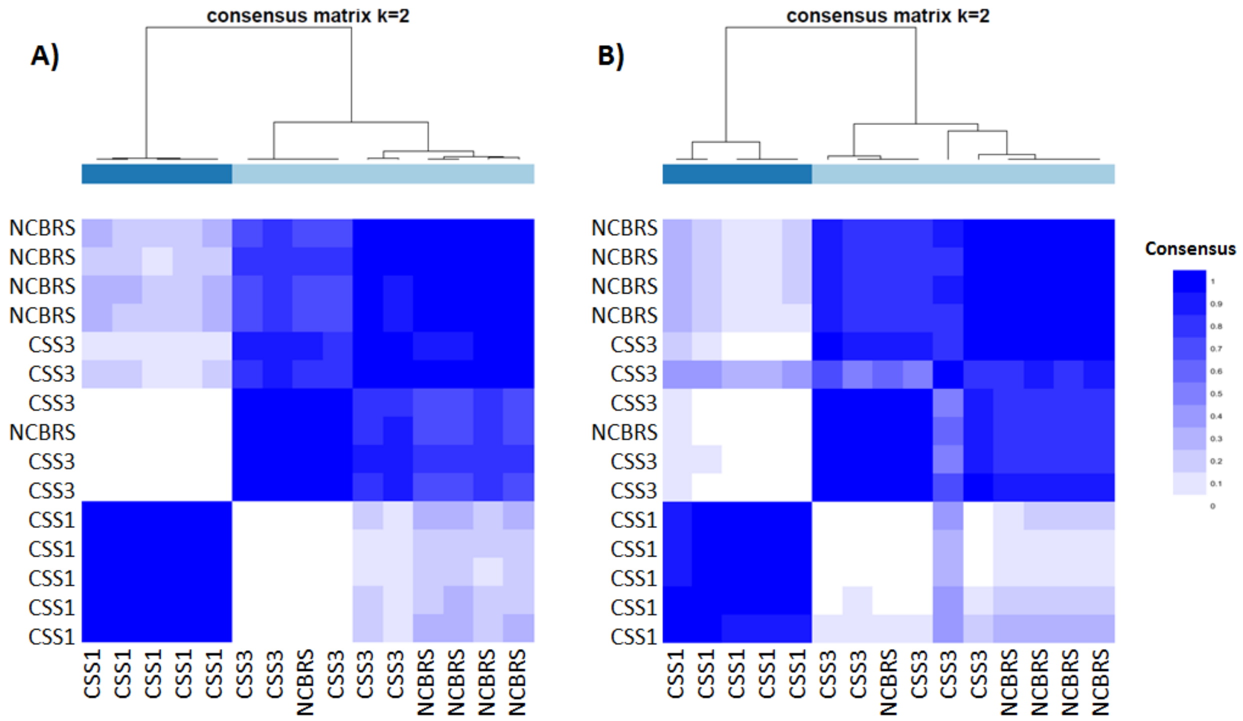

Supplementary Figure 21- Consensus clustering for equal sample sizes of CSS1, CSS3, and NCBRS (5 each)

Using the combination of the identified probes in the analysis of Figures S19 and S20 as well as the 135 probes found in CSS3, a consensus clustering was performed, as explained previously in Figure 3. This was only conducted for CSS1, CSS3, and NCBRS (n=15, five each) to ensure equal sample sizes in each three categories. The same result as the one in Figure 3 was observed, i.e., CSS3 and NCBRS cases always cluster together, whereas CSS1 generates a completely separate cluster of the two (section A). We next questioned whether this observation could be due to differences in probe counts, since the CSS3 and NCBRS have ~150 probes, whereas CSS1 has only 27. To control for this, we selected the top 150 probes sorted by p-value from the comparison of 5 CSS1 cases and 30 controls, and re-performed the analysis (section B). The same observation was noted, indicating that the similarity observed between CSS3 and NCBRS is not related to probe count or the sample size of the three subtypes.

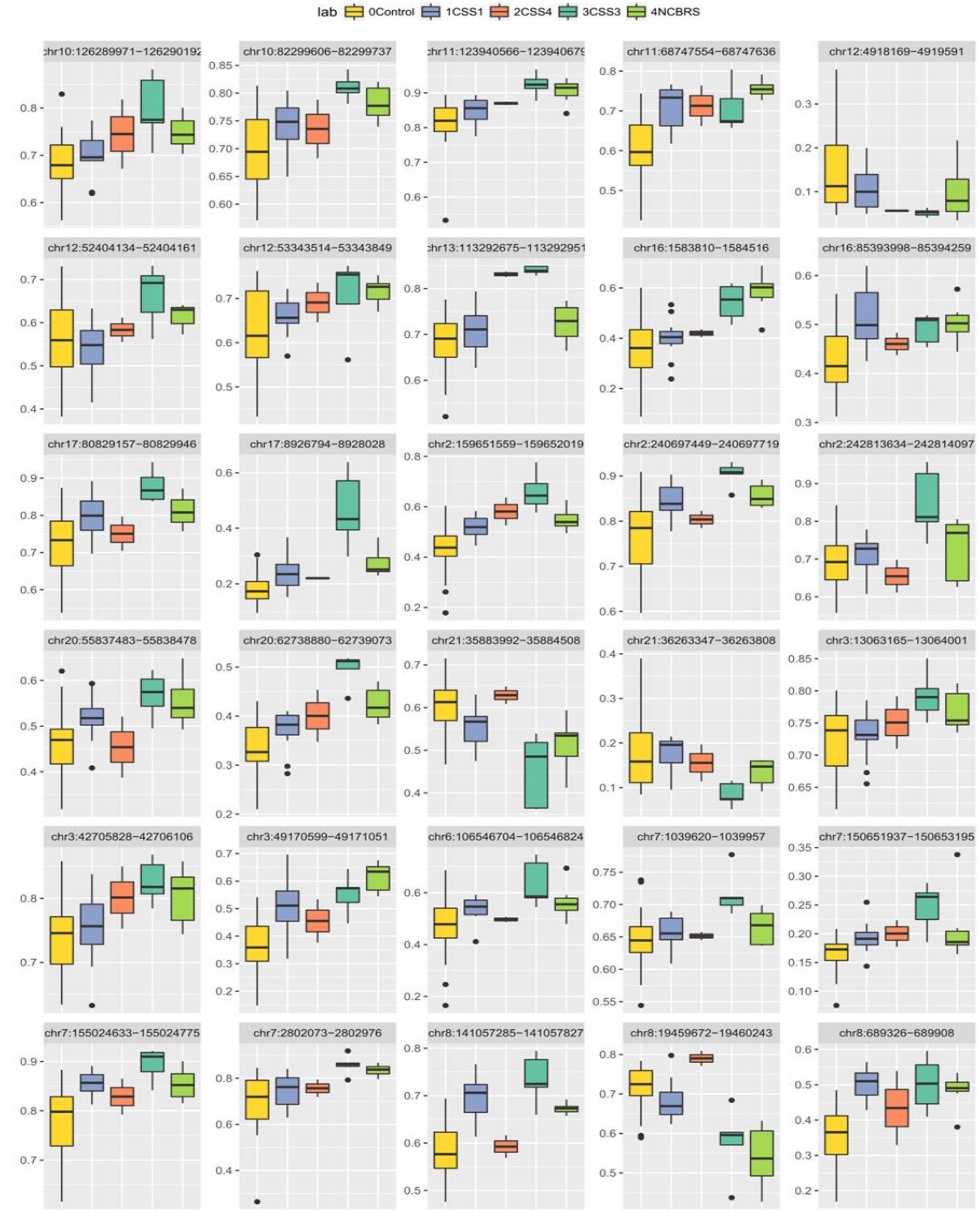

Supplementary Figure 22- DMRs differentially methylated in CSS3

The figure shows a total of 30 DMRs found to be differentially methylated in CSS cases compared to controls. The box plots represent the distribution of median methylation values across all of the probes mapping to each region stratified by the molecular class, i.e., Controls, CSS1, CSS4, CSS3, and NCBRS. These regions are detected in the comparison between CSS3 and controls; however, the majority of these regions in other CSS/NCBRS groups tend to show methylation values in the middle of a spectrum between controls and CSS3.Centre line: median of regional methylation levels across samples; Lower and upper bounds: first and third quartiles; Whiskers: Interquartile ranges

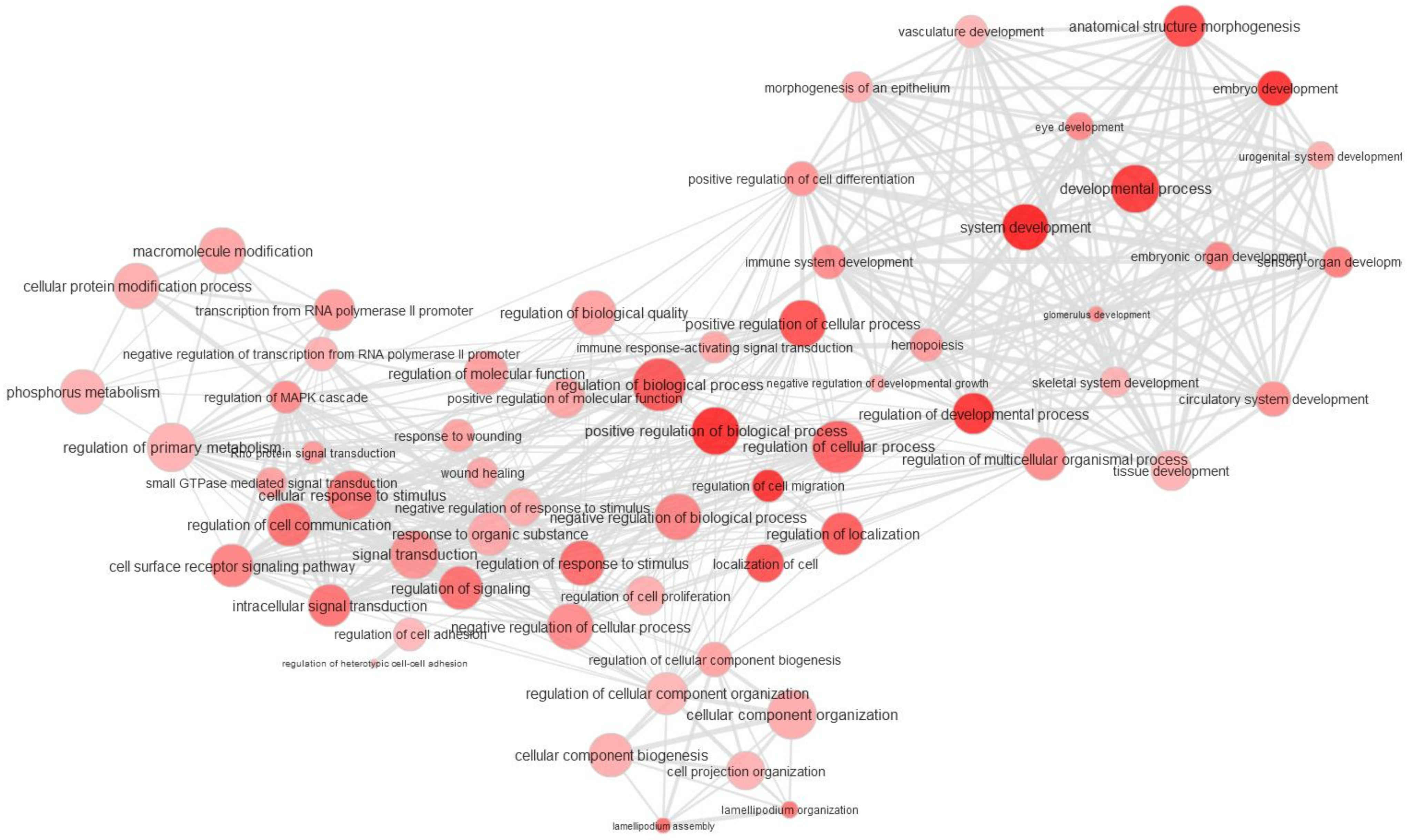

Supplementary Figure 23- Gene ontology terms enriched in the combined sets of CSS1, CSS3, and NCBRS methylation profiles

For details see Figure 7 in the manuscript.
