# Supplementary material for: BAFopathies’ DNA methylation epi-signatures demonstrate diagnostic utility and functional continuum of Coffin–Siris and Nicolaides–Baraitser syndromes
Source: Nat Commun. 2018 Nov 20;9:4885. doi: 10.1038/s41467-018-07193-y (PMC6244416; doi:10.1038/s41467-018-07193-y)
Supplement: Supplementary file 3 — Description of Additional Supplementary Files [file 41467_2018_7193_MOESM3_ESM.docx]

**Description of Additional Supplementary Files**

Supplementary Data 1: CpG sites differentially methylated in CSS1 (hg19)

Supplementary Data 2: CpG sites differentially methylated in CSS3 (hg19)

Supplementary Data 3: CpG sites differentially methylated in NCBRS (hg19)

Supplementary Data 4: DMRs found in three separate comparisons between CSS1, CSS3, and NCBRS and controls (hg19)

Supplementary Data 5: Gene ontology (GO) terms found in the three probe-sets

Supplementary Data 6: Gene ontology (GO) terms found in all three probe-sets

Supplementary Data 7: Details of the SVM classifier

Supplementary Data 8: Scores generated for different blood cells

Legend for Supplementary Data 7:

The model is trained on 131 features and 55 support vectors. Before prediction on a new database, the new methylation values have to be scaled according to the original database the model was trained on using the following equation:

Scaled CpG = (Unscaled CpG – Center) / scale

where CpG refers to the methylation beta value for every feature. Center and scale are provided in the table for each feature.

For prediction, the decision value of a new scaled data vector n is obtained by the following equation:

$$\sum_{i}^{n} a_{i}K\left( x_{i},n \right)+p$$

where x_i_ is the i-th support vector, a_i_ the corresponding coefficient, and K is the kernel function, all provided in the table. *Rho* (model intercept) in this equation is -0.4788158. The type of kernel used in this model is radial basis function (RBF) or Gaussian. The following is the RBF kernel equation for two vectors u and v:

K(u,v)=exp(-gamma*|u-v|^2)

The gamma hyperparameter to be used for kernel transformation is 0.007633588

Decision values > 0 indicate that the subject has the disease and decision values < 0 indicate otherwise. To convert these values into probability scores between 0-1, Platt’s scaling is performed:

P = 1/ (1+ exp (A.d + B))

where A and B represent the parameters of logistic distributions from the trained model (A= -5.212619 and B= 0.6499432). P is the probability of a screened subject for having a profile similar to CSS/NCBRS.
